# Distributed cooperative Kalman filter constrained by advection–diffusion equation for mobile sensor networks

**DOI:** 10.3389/frobt.2023.1175418

**Published:** 2023-06-07

**Authors:** Ziqiao Zhang, Scott T. Mayberry, Wencen Wu, Fumin Zhang

**Affiliations:** ^1^ School of Electrical and Computer Engineering, Georgia Institute of Technology, Atlanta, GA, United States; ^2^ George W. Woodruff School of Mechanical Engineering, Georgia Institute of Technology, Atlanta, GA, United States; ^3^ Computer Engineering Department of San Jose State University, San Jose, CA, United States; ^4^ Cheng Kar-Shun Robotics Institute, Hong Kong University of Science and Technology, Hong Kong, Hong Kong SAR, China

**Keywords:** distributed parameter systems, mobile sensor networks, Kalman filter, cooperative control, formation control

## Abstract

In this paper, a distributed cooperative filtering strategy for state estimation has been developed for mobile sensor networks in a spatial–temporal varying field modeled by the advection–diffusion equation. Sensors are organized into distributed cells that resemble a mesh grid covering a spatial area, and estimation of the field value and gradient information at each cell center is obtained by running a constrained cooperative Kalman filter while incorporating the sensor measurements and information from neighboring cells. Within each cell, the finite volume method is applied to discretize and approximate the advection–diffusion equation. These approximations build the weakly coupled relationships between neighboring cells and define the constraints that the cooperative Kalman filters are subjected to. With the estimated information, a gradient-based formation control law has been developed that enables the sensor network to adjust formation size by utilizing the estimated gradient information. Convergence analysis has been conducted for both the distributed constrained cooperative Kalman filter and the formation control. Simulation results with a 9-cell 12-sensor network validate the proposed distributed filtering method and control law.

## 1 Introduction

Natural phenomena, such as forest fires, hurricanes, and ocean eddies, are complex spatial–temporal processes that are influenced by physical parameters like wind speed, humidity, flow direction, flow speed, and temperature (Eshghi and Schmidtke, 2018; Wei et al., 2019; Chen and Dames, 2020; Park et al., 2018). By understanding these phenomena in real-time, precision countermeasures such as rerouting firefighters/AUVs to the growing edge of a forest fire, monitoring and evacuating local areas that are at risk of hurricane damage, or decreasing the search area of floating plane-crash survivors by measuring and estimating ocean eddies can be effectively deployed (Radmanesh et al., 2021; Elston et al., 2010). Partial differential equations (PDEs) have been proven to be effective in modeling many of these natural phenomena, and solving these PDEs can help us gain a better understanding of the spatial–temporal variations in these phenomena.

One typical PDE is the advection–diffusion equation, which has been applied to model a wide range of physical phenomena, including the propagation of chemicals, particles, energy, and other physical quantities inside media like water and air Ghez (2001). The advection–diffusion equation accounts for both spatial and temporal variations, making it applicable to many physical phenomena with varying parameters. Real-world applications of this equation include modeling oceanic oil spills like the Deepwater Horizon disaster in 2010 (Boufadel et al., 2022) and airborne chemical dispersion (Gao and Acar, 2016) resulting from train derailments, such as the one that occurred in Ohio in 2023. In both cases, understanding the behavior of the advection–diffusion equation can help predict the spread of pollutants and aid in the implementation of effective countermeasures.

Obtaining explicit analytic solutions for PDEs, such as the advection–diffusion equation, can be challenging in many cases. In the literature, static sensor networks have been proposed as a solution to estimation problems, where a large number of sensors are deployed over the domain of interest (Krstic and Smyshlyaev, 2008). However, static sensors have limited accuracy in areas with high spatial–temporal variations. To address this issue, mobile sensor networks have emerged as a more flexible and efficient alternative, requiring fewer sensors and enabling adaptive monitoring of areas of interest (Ge et al., 2019; Kumar et al., 2019; Wang et al., 2019).

In mobile sensor networks, measurements are collected along trajectories while the sensors move in groups within the field of interest. These mobile sensing data exhibit coupling between space and time, making it necessary to convert them into a map of spatial–temporal field estimates, instead of obtaining separate decoupled spatial and temporal maps Ge et al. (2019). To improve the accuracy of estimates, filters can be applied to combine multiple sensor measurements while accounting for measurement noise (Demetriou, 2021; Mishra et al., 2020; Hu et al., 2022).

Our previous work focused on solving the field estimation problem for mobile sensor networks in a centralized manner (Zhang and Leonard, 2010; You et al., 2022). Specifically, we derived information dynamics to model the spatial–temporal variations along the trajectory of the formation center of a cell of mobile sensors. We also proposed a cooperative Kalman filter to give estimations of the field value at formation center based on those derived information dynamics. In cases where the spatial–temporal field can be modeled by a known PDE, the PDE can be discretized as a constraint for the estimates of the cooperative Kalman filter (Wu et al., 2021; You et al., 2022). In our recent work (Zhang et al., 2022), we proposed a constrained cooperative Kalman filter and a framework to decouple the information dynamics and the PDE model.

In Zhang et al. (2023), we generalized the constrained cooperative Kalman filter to distributed mobile sensor networks with rigid formations in a spatial–temporal field modeled by the Poisson equation. In this approach, the mobile sensors are organized into cells and have limited communication capabilities restricted to intra-cell and adjacent cells. The estimated information at each cell center is shared only among neighboring cells according to the derived information dynamics.

The Kalman-consensus filter (Olfati-Saber, 2009) and its variants (Ji et al., 2017; Lian et al., 2020; Song et al., 2019; He et al., 2020) have been widely applied for distributed filtering, where a consensus term is added to the conventional Kalman filter for estimating states and reaching consensus with neighbors on the estimation. Different from these consensus-based filters, our proposed distributed filtering strategy does not focus on achieving consensus between neighboring sensors on the state estimation of targets. Instead, the information dynamics considered in this paper model the spatial–temporal variation along trajectories of cell centers and are defined based on the differences between neighbors. These differences establish the foundation of the proposed distributed filtering.

In this paper, we focus on solving the estimation problem for distributed mobile sensor networks in a field modeled by the advection–diffusion equation (Ghez, 2001). Distributed filtering over sensor networks has been studied with applications in target tracking, environmental monitoring, etc. Instead of maintaining all-to-all communication, sensors are only required to share information with their local neighbors, resulting in greater robustness and scalability compared with centralized filtering and estimation. In real-world scenarios such as underwater environments, low bandwidth, power limitations, high deployment expenses, and communication losses can make it challenging to maintain all-to-all communications among mobile sensor networks (Alfouzan, 2021; Omeke et al., 2021). This proposed distributed formulation is welcome in such real-world applications, as sensor power can be better distributed spatially. With limited communication, mobile sensors maintain a formation of organized cells with time-varying bounded relative distances while maneuvering in the field. For each cell, the field value and gradient information at the cell center are estimated using the sensor measurements from this cell.

The estimated information generated by the distributed filters is required to satisfy the advection–diffusion equation, which is used as a constraint for the estimation. Proper discretization and approximation of the continuous PDE are crucial in obtaining the estimation constraint. Finite volume methods are commonly applied to obtain PDE discretizations within each cell over the mesh grids composed of mobile sensors (Hermeline, 2000; Droniou, 2014). In the context of distributed mobile sensor networks, each node can be regarded as a grid point in a mesh grid that provides measurements of the field value at the grid point. The discretization of the advection–diffusion equation models the inclusion of sensor measurements in a single cell, with the information shared among neighboring cells. Such discretization is incorporated as constraints in the distributed cooperative Kalman filter and reflects the weakly coupled relationships among neighboring cells.

In this work, the desired relative positions between sensors are no longer constant and are determined by the estimated gradient information from the cooperative filters. In order to maintain the desired distances between sensors, a formation controller has been designed. Under the proposed formation control, the networked sensors are capable of spreading out with increased cell sizes when exploring fields with small gradients and forming a tighter size when the field has larger gradients. With adaptive formation sizes, the mobile sensors can explore larger areas in less time with minimal sacrifices to quality.

The major contributions of this paper are summarized as follows: 1) developing a distributed cooperative Kalman filter under constraints induced by the advection–diffusion equation for a mobile sensor network. 2) Applying the finite volume method for the PDE approximation. 3) Proving convergence for both the formation control and the distributed constrained cooperative Kalman filter.

The rest of the paper is organized as follows. Section 2 introduces the *Problem formulation*. Section 3 reviews the information dynamics and the measurement equations. Section 4 derives the approximation at each cell center using finite volume methods. Section 5 presents the distributed constrained cooperative Kalman filter for state estimation. Section 6 presents the formation control design for distributed mobile sensor networks. Section 7 provides the *Convergence analysis*. *Simulation results* are given in Section 8. *Conclusion and future works* follow in Section 9.

## 2 Problem formulation

Consider a spatial–temporal field in d-dimensional space, where $d\in Z_{+}$ and d ≥ 2. Denote the spatial domain of interest as $\Omega \subseteq R^{\text{d}}$ , location as ***r*** ∈ Ω, and time as $t\in R_{+}$ .

### 2.1 Environmental model and mobile sensors

We assume that the field can be described by the following advection–diffusion equation:

$$\frac{\partial z\left(r,t\right)}{\partial t}=\theta \Delta z\left(r,t\right)+v^{⊺}\nabla z\left(r,t\right),$$ (1)

where $z\left(r,t\right):R^{\text{d}}\times R_{+}\mapsto R$ is the field value that depends on both location ***r*** and time *t*, *θ* > 0 is a known constant diffusion coefficient, ∇ is the gradient operator, Δ = ∇^2^ represents the Laplacian operator, and $v\in R^{\text{d}}$ is a known constant vector representing flow velocity.

Eq. 1 has the initial condition *z*(***r***, 0) = r∈Ω and the boundary condition *z*(***r***, *t*) = *z*_*b*_(***r***, *t*) for ***r*** ∈ *∂*Ω, where *z*_0_(***r***) and *z*_*b*_(***r***, *t*) are arbitrary initial condition and Dirichlet boundary condition, respectively.

**FIGURE 1:**
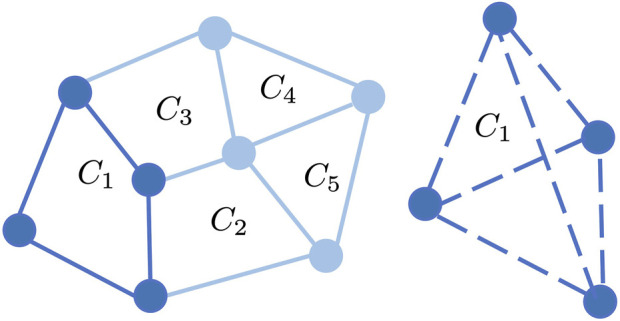
Example of five cells *C*_1_, …, *C*_5_ with eight sensors (left), where the blue dots denote mobile sensors and the solid lines represent boundary edges of each cell. Intra-*cell* communication of cell *C*_1_ (right), where the dashed lines represent communication between any two sensors from the same cell. Note that cells *C*_1_ and *C*_4_ do not share a boundary, and thus no communication occurs between these cells.

We suppose a number of distributed mobile sensors are taking discrete measurements of the field *z*(***r***, *t*), which conforms to the advection–diffusion equation described in Eq. 1. In a limited communication environment, these mobile sensors can only share information with their local neighbors and are unable to communicate globally. In this manner, the mobile sensors form communication *cells* with the ability to communicate intra-*cell* as well as with adjacent cells. No communication occurs between *cells* not sharing a boundary. The communication network of the mobile sensors is illustrated in Figure 1.

Assumption 2.1 The communication graph formed by the mobile sensors is predetermined and fixed, but the formation size changes while the sensors move in the field, as shown in Figure 1. The spatial domain covered by the mobile sensor network is defined as Ω_***r***_ with boundary *∂*Ω_***r***_, which both vary while the sensors move in the field.

Assumption 2.2 For each communication cell *C*_*j*_, the mobile sensors maintain distance from each other so that the area covered by the cell denoted by $A\left(C_{j}\right)$ will not shrink to zero size, i.e., $A\left(C_{j}\right)\neq 0$ for any *j* = 1, …, *N*.

Assumption 2.3 The mobile sensors communicate with neighboring sensors, and there is no overlap between any communication cells. The intersection between two cells is the edge connecting two vertices of the communication graph.

Definition 2.4 Two communication cells are called neighboring cells if they have a shared edge on the cell boundaries, e.g., *C*_1_, *C*_2_ in Figure 1.

Assumption 2.5 Every communication cell has at least one neighboring cell. The sensors maintain all-to-all information sharing within each communication cell. The information sharing among different communication cells only happens between neighboring cells, and the shared information is the corresponding estimated information at cell centers.

We suppose the mobile sensors form multiple communication cells *C*_1_, *C*_2_, …, *C*_*N*_. (The communication cells will be denoted as cells for simplicity in the rest of this paper.) Denote the *i*th mobile sensor from cell *C*_*j*_ as (*i*, *j*) and the location of this mobile sensor at the *k*th time step as $r_{i,j}^{k}$ . Then, the corresponding noisy measurement $p\left(r_{i,j}^{k},k\right)$ taken by this sensor can be written as

$$p\left(r_{i,j}^{k},k\right)=z\left(r_{i,j}^{k},k\right)+n_{i,j}^{k},$$ (2)

where $n_{i,j}^{k}\in R$ is an i.i.d. Gaussian noise with zero mean. Define the center location of sensors from cell *C*_*j*_ at the *k*th time step as $r_{c_{j}}^{k}$ and $r_{c_{j}}^{k}=\frac{1}{\left|C_{j}\right|}\sum_{i\in C_{j}}r_{i,j}^{k}$ , where |*C*_*j*_| is the number of mobile sensors belonging to cell *C*_*j*_.

### 2.2 Formation control

We assume that the desired distance $d_{i_{1},i_{2}}^{k}$ at the *k*th time step between any two communicating sensors *i*_1_, *i*_2_ from the same communication cell is non-constant and can be determined by gradient information as follows:

$$d_{i_{1},i_{2}}^{k}=d\left(\nabla z\left(r_{i_{1}}^{k},k\right),\nabla z\left(r_{i_{2}}^{k},k\right)\right),$$ (3)

where function $d\left(\cdot \right):R^{\text{d}}\mapsto R$ describes the relationship between the desired distance and gradient information at the cell center $r_{c_{j}}$ at the *k*th time step.

The velocity of sensor *i* from communication cell *C*_*j*_ is described by

$${\dot{r}}_{i,j}^{k}=u\left(z\left(r_{i,j}^{k}\right),\nabla z\left(r_{i,j}^{k}\right),d_{i}^{k}\right),$$ (4)

where ***u*** is a control law that relies on the measurement *z*(***r***_*i*_), gradient ∇*z*(***r***_*i*_) of the field function, and the desired distance vector $d_{i}^{k}={\left[d_{i,i^{\prime }}^{k}\right]}_{i^{\prime }\in N_{i}}$ containing all desired distance information of sensor *i*.

### 2.3 Design goals

By utilizing the discrete measurements taken by mobile sensors, we would like to solve the estimation problem for distributed mobile sensor networks in a field modeled by the advection–diffusion equation. While the sensors are deployed in the field collecting measurements, the estimated information will be applied to maintain desired formations and guide them moving in the field.

The objectives of this paper can be summarized as follows: 1) estimate field and gradient information at cell centers along trajectories by incorporating discrete sensor measurements. 2) Develop a formation controller such that the sensors will move in desired varying formation sizes by incorporating the estimated gradient information while moving in the field of interest. 3) Provide detailed convergence analysis of the estimation under varying formations. 4) Apply formation control to the distributed sensor network to explore the field utilizing estimated information.

## 3 Preliminaries

In this section, we will review the results of information dynamics and measurement equations at each cell center. For more detailed derivation, interested readers can refer to our previous papers (Zhang et al., 2022) for information dynamics and (Zhang et al., 2023) for distributed approximation using the finite volume method.

### 3.1 Information dynamics

The relationship between total derivative $\frac{dz}{dt}$ and partial derivative $\frac{\partial z}{\partial t}$ of $r_{c_{j}}^{k}$ at the *k*th time step can be approximated using the chain rule as follows:

$${\left.\left(\frac{dz}{dt}-\frac{\partial z}{\partial t}\right)\right|}_{\left(r_{c_{j}}^{k},k\right)}\approx \frac{1}{\delta t}{\left(r_{c_{j}}^{k}-r_{c_{j}}^{k-1}\right)}^{⊺}\nabla z\left(r_{c_{j}}^{k-1},k-1\right),$$ (5)

where *δt* is the sampling time interval. By applying the finite difference method, we can obtain $\frac{dz}{dt}$ and $\frac{\partial z}{\partial t}$ as

$${\left.\frac{dz}{dt}\right|}_{\left(r_{c_{j}}^{k},k\right)}\approx \frac{z\left(r_{c_{j}}^{k},k\right)-z\left(r_{c_{j}}^{k-1},k-1\right)}{\delta t},\;{\left.\frac{\partial z}{\partial t}\right|}_{\left(r_{c_{j}}^{k},k\right)}\approx \frac{z\left(r_{c_{j}}^{k},k+1\right)-z\left(r_{c_{j}}^{k},k\right)}{\delta t},$$ (6)

which leads to

$${\left.\left(\frac{dz}{dt}-\frac{\partial z}{\partial t}\right)\right|}_{\left(r_{c_{j}}^{k},k\right)}\approx \frac{1}{\delta t}\left(2z\left(r_{c_{j}}^{k},k\right)-z\left(r_{c_{j}}^{k-1},k-1\right)-z\left(r_{c_{j}}^{k},k+1\right)\right).$$ (7)

From Eqs 5, 7, we can build the following relationship:

$$\left(2z\left(r_{c_{j}}^{k},k\right)-z\left(r_{c_{j}}^{k-1},k-1\right)-z\left(r_{c_{j}}^{k},k+1\right)\right)={\left(r_{c_{j}}^{k}-r_{c_{j}}^{k-1}\right)}^{⊺}\nabla z\left(r_{c_{j}}^{k-1},k-1\right).$$ (8)

Since $z\left(r_{c_{j}}^{k},k\right)=z\left(r_{c_{j}}^{k-1},k\right)+{\left(r_{c_{j}}^{k}-r_{c_{j}}^{k-1}\right)}^{⊺}\nabla z\left(r_{c_{j}}^{k-1},k\right)$ , we should have

$$\begin{array}{cc} z\left(r_{c_{j}}^{k},k+1\right)= & 2z\left(r_{c_{j}}^{k},k\right)-z\left(r_{c_{j}}^{k-1},k-1\right)\\ & -{\left(r_{c_{j}}^{k}-r_{c_{j}}^{k-1}\right)}^{⊺}\nabla z\left(r_{c_{j}}^{k-1},k-1\right)\\ = & 2z\left(r_{c_{j}}^{k-1},k\right)+2{\left(r_{c_{j}}^{k}-r_{c_{j}}^{k-1}\right)}^{⊺}\nabla z\left(r_{c_{j}}^{k-1},k\right)\\ & -z\left(r_{c_{j}}^{k-1},k-1\right)\\ & -{\left(r_{c_{j}}^{k}-r_{c_{j}}^{k-1}\right)}^{⊺}\nabla z\left(r_{c_{j}}^{k-1},k-1\right). \end{array}$$ (9)

By utilizing a similar approach, we can obtain

$$\begin{array}{ll} {\left.\frac{dz}{dt}\right|}_{\left(r_{c_{j}}^{k},k+1\right)} & \approx \frac{z\left(r_{c_{j}}^{k},k+1\right)-z\left(r_{c_{j}}^{k-1},k\right)}{\delta t},\\ {\left.\;\frac{\partial z}{\partial t}\right|}_{\left(r_{c_{j}}^{k},k+1\right)} & \approx \frac{z\left(r_{c_{j}}^{k},k+1\right)-z\left(r_{c_{j}}^{k},k\right)}{\delta t}, \end{array}$$ (10)

leading to

$$\begin{array}{ll} {\left.\left(\frac{dz}{dt}-\frac{\partial z}{\partial t}\right)\right|}_{\left(r_{c_{j}}^{k},k+1\right)} & \approx \frac{1}{\delta t}\left(z\left(r_{c_{j}}^{k},k\right)-z\left(r_{c_{j}}^{k-1},k\right)\right)\\ & =\frac{1}{\delta t}{\left(r_{c_{j}}^{k}-r_{c_{j}}^{k-1}\right)}^{⊺}\nabla z\left(r_{c_{j}}^{k-1},k\right), \end{array}$$ (11)

which means that

$$z\left(r_{c_{j}}^{k},k\right)-z\left(r_{c_{j}}^{k-1},k\right)={\left(r_{c_{j}}^{k}-r_{c_{j}}^{k-1}\right)}^{⊺}\nabla z\left(r_{c_{j}}^{k-1},k\right).$$ (12)

We define the state variable $x\left(j,k\right)=\left[z\left(r_{c_{j}}^{k-1},k-1\right),\nabla z\left(r_{c_{j}}^{k-1},k-1\right),\right.{\left.z\left(r_{c_{j}}^{k-1},k\right),\nabla z\left(r_{c_{j}}^{k-1},k\right)\right]}^{⊺}$ , and we can get the following state equation based on Eqs 9, 12:

$$x\left(j,k+1\right)=A\left(j,k\right)x\left(j,k\right)+U\left(j,k\right)+e\left(j,k\right),$$ (13)

where $A\left(j,k\right)≜\left[\begin{array}{cccc} 0 & 0 & 1 & {\left(r_{c_{j}}^{k}-r_{c_{j}}^{k-1}\right)}^{⊺}\\ 0 & I_{\text{d}\times d} & 0 & 0\\ -1 & -{\left(r_{c_{j}}^{k}-r_{c_{j}}^{k-1}\right)}^{⊺} & 2 & 2{\left(r_{c_{j}}^{k}-r_{c_{j}}^{k-1}\right)}^{⊺}\\ 0 & 0 & 0 & I \end{array}\right]$ , $U\left(j,k\right)≜\left[\begin{array}{c} 0\\ \nabla^{2}z\left(r_{c_{j}}^{k-1},k-1\right)\left(r_{c_{j}}^{k}-r_{c_{j}}^{k-1}\right)\\ 0\\ \nabla^{2}z\left(r_{c_{j}}^{k-1},k\right)\left(r_{c_{j}}^{k}-r_{c_{j}}^{k-1}\right) \end{array}\right]$ , and ***e*** (*j*, *k*) is the noise term.

### 3.2 Measurement equation

The field concentration can be locally approximated by the Taylor series up to the second order as

$$\begin{array}{cc} z\left(r_{i,j}^{k-1},k-1\right)\approx & z\left(r_{c_{j}}^{k-1},k-1\right)+{\left(r_{i,j}^{k-1}-r_{c_{j}}^{k-1}\right)}^{⊺}\nabla z\left(r_{c_{j}}^{k-1},k-1\right)\\ & +\frac{1}{2}{\left(r_{i,j}^{k-1}-r_{c_{j}}^{k-1}\right)}^{⊺}H\left(r_{c_{j}}^{k-1},k-1\right)\left(r_{i,j}^{k-1}-r_{c_{j}}^{k-1}\right),\\ z\left(r_{i,j}^{k},k\right)\approx & z\left(r_{c_{j}}^{k-1},k\right)+{\left(r_{i,j}^{k}-r_{c_{j}}^{k-1}\right)}^{⊺}\nabla z\left(r_{c_{j}}^{k-1},k\right)\\ & +\frac{1}{2}{\left(r_{i,j}^{k}-r_{c_{j}}^{k-1}\right)}^{⊺}H\left(r_{c_{j}}^{k-1},k-1\right)\left(r_{i,j}^{k}-r_{c_{j}}^{k-1}\right). \end{array}$$ (14)

Let $Z\left(j,k\right)={\left[z\left(r_{1,j}^{k-1},k-1\right),\ldots ,z\left(r_{|C_{j}|,j}^{k-1},k-1\right),z\left(r_{1,j}^{k},k\right),\ldots ,\;z\left(r_{|C_{j}|,j}^{k},k\right)\right]}^{⊺}$ be the vector of true field values. Define

$$\begin{array}{ll} C\left(j,k\right) & ≜\left[\begin{array}{cccc} 1 & {\left(r_{1,j}^{k-1}-r_{c_{j}}^{k-1}\right)}^{⊺} & 0 & 0\\ \vdots & \vdots & \vdots & \vdots \\ 1 & {\left(r_{|C_{j}|,j}^{k-1}-r_{c_{j}}^{k-1}\right)}^{⊺} & 0 & 0\\ 0 & 0 & 1 & {\left(r_{1,j}^{k}-r_{c_{j}}^{k-1}\right)}^{⊺}\\ \vdots & \vdots & \vdots & \vdots \\ 0 & 0 & 1 & {\left(r_{|C_{j}|,j}^{k}-r_{c_{j}}^{k-1}\right)}^{⊺} \end{array}\right],\\ D\left(j,k\right) & ≜\left[\begin{array}{c} \frac{1}{2}{\left[\left(r_{1,j}^{k-1}-r_{c_{j}}^{k-1}\right)⊗\left(r_{1,j}^{k-1}-r_{c_{j}}^{k-1}\right)\right]}^{⊺}\\ \vdots \\ \frac{1}{2}{\left[\left(r_{|C_{j}|,j}^{k-1}-r_{c_{j}}^{k-1}\right)⊗\left(r_{|C_{j}|,j}^{k-1}-r_{c_{j}}^{k-1}\right)\right]}^{⊺}\\ \frac{1}{2}{\left[\left(r_{1,j}^{k}-r_{c_{j}}^{k-1}\right)⊗\left(r_{1,j}^{k}-r_{c_{j}}^{k-1}\right)\right]}^{⊺}\\ \vdots \\ \frac{1}{2}{\left[\left(r_{|C_{j}|,j}^{k}-r_{c_{j}}^{k-1}\right)⊗\left(r_{|C_{j}|,j}^{k}-r_{c_{j}}^{k-1}\right)\right]}^{⊺} \end{array}\right], \end{array}$$ (15)

where *⊗* is the Kronecker product. The Taylor expansions (Eq. 14) for all sensors near $r_{c_{j}}^{k-1}$ can be rewritten in a vector form as

$$Z\left(j,k\right)=C\left(j,k\right)x\left(j,k\right)+D\left(j,k\right)H\left(j,k\right),$$ (16)

where ***H*** (*j*, *k*) is a column vector obtained by rearranging elements of the Hessian $H\left(r_{c_{j}}^{k-1},k-1\right)$ .

Supposing $\hat{H}\left(j,k\right)$ represents the estimate of the vector form Hessian ***H*** (*j*, *k*) at $r_{c_{j}}^{k}$ , Eq. 2 can be remodeled as

$$P\left(j,k\right)=C\left(j,k\right)x\left(j,k\right)+D\left(j,k\right)\hat{H}\left(j,k\right)+D\left(j,k\right)\varepsilon \left(j,k\right)+n\left(j,k\right),$$ (17)

where $P\left(j,k\right)=\left[p\left(r_{1,j}^{k-1},k-1\right),\ldots ,p\left(r_{|C_{j}|,j}^{k-1},k-1\right),p\left(r_{1,j}^{k},k\right),\ldots ,\right.{\left.p\left(r_{|C_{j}|,j}^{k},k\right)\right]}^{⊺}$ is the measurement vector, ***ɛ*** (*j*, *k*) represents the error in Hessian estimation, and **n**(*j*, *k*) is the vector of Gaussian noise *n*_*i*_ in Eq. 2.

If ***D*** (*j*,*k*)^⊺^ ***D*** (*j*, *k*) is invertible, then the least mean square method can be applied to approximate the Hessian vector as follows (You et al., 2022):

$$\hat{H}\left(j,k\right)={\left(D{\left(j,k\right)}^{T}D\left(j,k\right)\right)}^{-1}D{\left(j,k\right)}^{T}\left[P\left(j,k\right)-C\left(j,k\right)x\left(j,k\right)\right].$$ (18)

According to the definition of ***D*** (*j*, *k*) in Eq. 15, at least d^2^ linearly independent vectors of $\left(r_{i,j}^{k-1}-r_{c_{j}}^{k-1}\right),\left(r_{i,j}^{k}-r_{c_{j}}^{k-1}\right)$ are needed for invertible ***D*** (*j*,*k*)^⊺^ ***D*** (*j*, *k*), which means 2|*C*_*j*_|≥d^2^. In the case of d = 2, |*C*_*j*_| should satisfy that $|C_{j}|\geq \frac{d^{2}}{2}=2$ . Since |*C*_*j*_| = 2 means two sensors in one cell leading to $A\left(C_{j}\right)=0$ , which violates $A\left(C_{j}\right)\neq 0$ , at least three sensors are needed to form one cell.

If ***D*** (*j*,*k*)^⊺^ ***D*** (*j*, *k*) is not invertible, then a regularization term *ϵ*_0_ *I* will be added as follows:

$$\hat{H}\left(j,k\right)={\left(D{\left(j,k\right)}^{T}D\left(j,k\right)+\epsilon_{0}I\right)}^{-1}D{\left(j,k\right)}^{T}\left[P\left(j,k\right)-C\left(j,k\right)x\left(j,k\right)\right],$$ (19)

where *ϵ*_0_ > 0 is a small positive scalar and $I\in R^{\text{d}\times d}$ is the identity matrix.

## 4 Approximation using the finite volume method

Since the measurements taken by the mobile sensors in Eq. 2 are discrete, the continuous advection–diffusion equation in Eq. 1 should be discretized properly. The mobile sensors have already been organized into different cells, and the finite volume method can be applied for PDE discretization within each cell.

The advection–diffusion equation in Eq. 1 can be discretized at $r_{c_{j}}^{k-1}$ at the (*k* − 1)-th time step as follows:

$$\frac{z\left(r_{c_{j}}^{k-1},k\right)-z\left(r_{c_{j}}^{k-1},k-1\right)}{\Delta t}=\theta \Delta z\left.\left(r_{c_{j}}^{k-1},k-1\right)+v^{⊺}\nabla z\left(r_{c_{j}}^{k-1},k-1\right)\right).$$ (20)

As we can observe that $z\left(r_{c_{j}}^{k-1},k\right),z\left(r_{c_{j}}^{k-1},k-1\right),\nabla z\left(r_{c_{j}}^{k-1},k-1\right)$ are elements in the state variable ***x*** (*j*, *k*), the only term left is the Laplacian $\Delta z\left(r_{c_{j}}^{k-1},k-1\right)$ . We consider using the finite volume method to obtain the Laplacian approximation such that the discretized advection–diffusion equation can be utilized as a constraint for the distributed Kalman filter.

**FIGURE 2:**
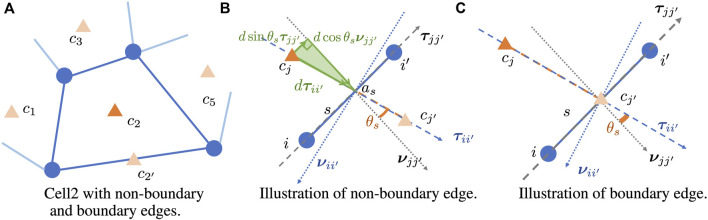
Illustration of ***ν***_*jj*′_, ***τ***_*jj*′_, ***ν***_*ii*′_, ***τ***_*ii*′_, *θ*_*s*_ with given cell centers *c*_*j*_, *c*_*j*′_ and given edge *s* connecting vertices *i*, *i*′. Orange triangles represent cell centers. **(A)** Cell 2 with non-boundary and boundary edges. **(B)** Illustration of non-boundary edge. **(C)** Illustration of boundary edge.

For each formation of sensors belonging to the same communication cell, the finite volume method in Hermeline (2000) will be applied to generate the approximation. We will present the general results covering both non-boundary edge and boundary edge cases, as illustrated in Figure 2A. Since all the values are at the same *k*th time step, we will drop the time index *k* for simplicity in this approximation.

Consider a shared edge *s* by *C*_*j*_ and neighboring cell *C*_*j*′_. Denote the cell centers for *C*_*j*_, *C*_*j*′_ as *c*_*j*_, *c*_*j*′_, respectively. Denote the two vertices of edge *s* as *i*, *i*′. As shown in Figure 2B, denote ***ν***_*jj*′_ as the unit outward normal vector on edge *s* connecting *i* and *i*′, with ***τ***_*jj*′_ as the corresponding unit counterclockwise tangent vector, and denote ***ν***_*ii*′_ as the unit outward normal vector on edge connecting *c*_*j*_ and *c*_*j*′_, with ***τ***_*ii*′_ as the corresponding unit counterclockwise tangent vector. Define *θ*_*s*_ to be the angle between ***ν***_*jj*′_ and ***τ***_*ii*′_. Suppose the edge connecting *c*_*j*_ and *c*_*j*′_ intersects with edge *s* at point *a*_*s*_. Denote the length of the edge connecting *c*_*j*_ and *a*_*s*_ as *d*. Then, the three vectors *d* ***τ***_*ii*′_, *d* cos *θ*_*s*_ ***ν***_*jj*′_, *d* sin *θ*_*s*_ ***τ***_*jj*′_ form a right triangle, as shown by the green shaded area in Figure 2B. This leads to the following relationship:

$$\nu_{jj^{\prime }}=-\tan\theta_{s}\tau_{jj^{\prime }}+\frac{1}{\cos\theta_{s}}\tau_{ii^{\prime }}.$$ (21)

If there is no such neighboring cell *C*_*j*′_, then the middle point of edge *s* can be treated as *c*_*j*′_, as illustrated in Figure 2C. By taking integrals of Δ*z* over cell *C*_*j*_, we can obtain $\int \int_{C_{j}}\Delta z=\sum_{s\in \partial C_{j}}\int_{S_{ii^{\prime }}}\nabla z\cdot \nu_{jj^{\prime }}$ . Since $A\left(C_{j}\right)\neq 0$ , substituting ***ν***_*jj*′_ with Eq. 21 leads to21 leads to

$$\begin{array}{cc} \Delta z\approx \frac{1}{A\left(C_{j}\right)}\iint_{C_{j}}\Delta z= & \frac{1}{A\left(C_{j}\right)}\underset{s\in \partial C_{j}}{\sum }\int_{S_{ii^{\prime }}}\nabla z\cdot \nu_{jj^{\prime }}\\ = & \frac{1}{A\left(C_{j}\right)}\underset{s\in \partial C_{j}}{\sum }\left[-\tan\theta_{s}\int_{S_{ii^{\prime }}}\nabla z\cdot \tau_{jj^{\prime }}\right.\\ & \left.+\frac{1}{\cos\theta_{s}}\int_{S_{ii^{\prime }}}\nabla z\cdot \tau_{ii^{\prime }}\right]. \end{array}$$ (22)

By the finite difference method, we know that

$$\begin{array}{cc} \int_{S_{ii^{\prime }}}\nabla z\cdot \tau_{jj^{\prime }} & \approx z_{i}-z_{i^{\prime }},\\ \int_{S_{ii^{\prime }}}\nabla z\cdot \tau_{ii^{\prime }} & \approx \left\{\begin{array}{cc} & \frac{\|r_{i,j}-r_{i^{\prime },j}{\|}_{2}}{\|r_{c_{j}}-r_{c_{j^{\prime }}}{\|}_{2}}\left(z_{c_{j^{\prime }}}-z_{c_{j}}\right),\text{ if }S_{ii^{\prime }}\notin \partial \Omega_{r},\\ & \frac{\|r_{i,j}-r_{i^{\prime },j}{\|}_{2}}{\|r_{c_{j}}-r_{c_{j^{\prime }}}{\|}_{2}}\left(\frac{z_{i}+z_{i^{\prime }}}{2}-z_{c_{j}}\right),\text{ if }S_{ii^{\prime }}\in \partial \Omega_{r}, \end{array}\right. \end{array}$$ (23)

where *S*_*ii*′_ is the edge connecting mobile sensors *i*, *i*′, and *∂*Ω_***r***_ is the boundary of the area covered the entire mobile sensor network. This will lead to

$$\begin{array}{cc} \Delta z= & -\frac{1}{A\left(C_{j}\right)}\underset{s\in \partial C_{j}}{\sum }\tan\theta_{s}\left(z_{i}-z_{i^{\prime }}\right)\\ & +\frac{1}{A\left(C_{j}\right)}\underset{s\in \left(\partial C_{j}⧵\partial \Omega_{r}\right)}{\sum }\frac{1}{\cos\theta_{s}}\frac{\|r_{i,j}-r_{i^{\prime },j}{\|}_{2}}{\|r_{c_{j}}-r_{c_{j^{\prime }}}{\|}_{2}}\left(z_{c_{j^{\prime }}}-z_{c_{j}}\right)\\ & +\frac{1}{A\left(C_{j}\right)}\underset{s\in \left(\partial C_{j}\cap \partial \Omega_{r}\right)}{\sum }\frac{1}{\cos\theta_{s}}\frac{\|r_{i,j}-r_{i^{\prime },j}{\|}_{2}}{\|r_{c_{j}}-r_{c_{j^{\prime }}}{\|}_{2}}\\ & \times \left(\frac{z_{i}+z_{i^{\prime }}}{2}-z_{c_{j}}\right). \end{array}$$ (24)

Note that if cell *C*_*j*_ has no boundary edges, then *∂C*_*j*_⧵*∂*Ω_***r***_ = *∂C*_*j*_ and *∂C*_*m*_ ∩ *∂*Ω_***r***_ = ∅. Thus, the approximation in Eq. 24 covers the cases with and without boundary edges. Substituting Δ*z* in Eq. 24 back to the discretized advection–diffusion (Eq. 20) will lead to

$$\begin{array}{cc} & \frac{z\left(r_{c_{j}}^{k-1},k\right)-z\left(r_{c_{j}}^{k-1},k-1\right)}{\Delta t}=v^{⊺}\nabla z\left(r_{c_{j}}^{k-1},k-1\right)\\ & \;-\frac{\theta }{A\left(C_{j}\right)}\underset{s\in \partial C_{j}}{\sum }\tan\theta_{s}\left[z\left(r_{i,j}^{k-1},k-1\right)-z\left(r_{i^{\prime },j}^{k-1},k-1\right)\right]\\ & \;+\frac{\theta }{A\left(C_{j}\right)}\underset{s\in \left(\partial C_{j}⧵\partial \Omega_{r}\right)}{\sum }\frac{1}{\cos\theta_{s}}\frac{\|r_{i,j}^{k-1}-r_{i^{\prime },j}^{k-1}{\|}_{2}}{\|r_{c_{j}}^{k-1}-r_{c_{j^{\prime }}}^{k-1}{\|}_{2}}\\ & \;\times \left[z\left(r_{c_{j^{\prime }}}^{k-1},k-1\right)-z\left(r_{c_{j}}^{k-1},k-1\right)\right]\\ & \;+\frac{\theta }{A\left(C_{j}\right)}\underset{s\in \left(\partial C_{j}\cap \partial \Omega_{r}\right)}{\sum }\frac{1}{\cos\theta_{s}}\frac{\|r_{i,j}^{k-1}-r_{i^{\prime },j}^{k-1}{\|}_{2}}{\|r_{c_{j}}^{k-1}-r_{c_{j^{\prime }}}^{k-1}{\|}_{2}}\\ & \;\times \left[\frac{z\left(r_{i,j}^{k-1},k-1\right)+z\left(r_{i^{\prime },j}^{k-1},k-1\right)}{2}-z\left(r_{c_{j}}^{k-1},k-1\right)\right]. \end{array}$$ (25)

This can be rewritten in linear form with respect to state variable $x\left(j,k\right)={\left[z\left(r_{c_{j}}^{k-1},k-1\right),\nabla z\left(r_{c_{j}}^{k-1},k-1\right),z\left(r_{c_{j}}^{k-1},k\right),\nabla z\left(r_{c_{j}}^{k-1},k\right)\right]}^{⊺}$

$$G^{⊺}\left(j,k\right)x\left(j,k\right)=g\left(j,k\right),$$ (26)

where $G\left(j,k\right)≜{\left[\begin{array}{cccc} \left(-1+\frac{\theta \Delta t}{A\left(C_{j}\right)}\sum_{s\in \partial C_{j}}\frac{1}{\cos\theta_{s}}\frac{\|r_{i,j}^{k-1}-r_{i^{\prime },j}^{k-1}{\|}_{2}}{\|r_{c_{j}}^{k-1}-r_{c_{j^{\prime }}}^{k-1}{\|}_{2}}\right) & -{\left(v\Delta t\right)}^{⊺} & 1 & 0_{1\times d} \end{array}\right]}^{⊺}$ , and

$$\begin{array}{cc} g\left(j,k\right)≜ & -\frac{\theta \Delta t}{A\left(C_{j}\right)}\underset{s\in \partial C_{j}}{\sum }\tan\theta_{s}\left[z\left(r_{i,j}^{k-1},k-1\right)-z\left(r_{i^{\prime },j}^{k-1},k-1\right)\right]\\ & +\frac{\theta \Delta t}{A\left(C_{j}\right)}\underset{s\in \left(\partial C_{j}⧵\partial \Omega_{r}\right)}{\sum }\frac{1}{\cos\theta_{s}}\frac{\|r_{i,j}^{k-1}-r_{i^{\prime },j}^{k-1}{\|}_{2}}{\|r_{c_{j}}^{k-1}-r_{c_{j^{\prime }}}^{k-1}{\|}_{2}}z\left(r_{c_{j^{\prime }}}^{k-1},k-1\right)\\ & +\frac{\theta \Delta t}{A\left(C_{j}\right)}\underset{s\in \left(\partial C_{j}\cap \partial \Omega_{r}\right)}{\sum }\frac{1}{\cos\theta_{s}}\frac{\|r_{i,j}^{k-1}-r_{i^{\prime },j}^{k-1}{\|}_{2}}{\|r_{c_{j}}^{k-1}-r_{c_{j^{\prime }}}^{k-1}{\|}_{2}}\frac{z\left(r_{i,j}^{k-1},k-1\right)+z\left(r_{i^{\prime },j}^{k-1},k-1\right)}{2}. \end{array}$$ (27)

## 5 State estimation using constrained cooperative Kalman filter

In this section, we will present the distributed constrained cooperative Kalman filter, which uses local sensor measurements to estimate information at each cell center and treats the PDE model as a constraint for the estimated information.

Assumption 5.1 We assume that the noises ***e*** (*j*, *k*), ***ɛ*** (*j*, *k*), and **n**(*j*, *k*) are i.i.d. Gaussian noise with zero mean and with constant covariance matrix for all *j*, i.e., E[***e*** (*j*, *k*) ***e*** ^⊺^(*j*, *k*)] = ***W***, E[***ɛ*** (*j*, *k*) ***ɛ*** ^⊺^(*j*, *k*)] = ***Q***, and E[**n**(*j*, *k*)**n** ^⊺^(*j*, *k*)] = ***R***_*n*_.

The constrained cooperative Kalman filter can be constructed using six steps:

(1) One-step state prediction

$${\hat{x}}^{-}\left(j,k\right)=A\left(j,k-1\right){\tilde{x}}^{+}\left(j,k-1\right)+U\left(j,k-1\right),$$ (28)

where ${\tilde{x}}^{+}\left(j,k-1\right)$ is the constrained state estimate from the previous time step and ${\hat{x}}^{-}\left(j,k\right)$ is the one-step state prediction.

(2) Error covariance of ${\hat{x}}^{-}\left(j,k\right)$

$$R^{-}\left(j,k\right)=A\left(j,k-1\right)R^{+}\left(j,k-1\right)A^{⊺}\left(j,k-1\right)+W.$$ (29)

(3) Optimal gain

$$\begin{array}{ll} K\left(j,k\right) & =R^{-}\left(j,k\right)C^{⊺}\left(j,k\right)\left[C\left(j,k\right)R^{-}\left(j,k\right)C^{⊺}\left(j,k\right)\right.\\ & \;+{\left.D\left(j,k\right)QD^{⊺}\left(j,k\right)+R_{n}\right]}^{-1}. \end{array}$$ (30)

The Hessian estimate can be approximated using the one-step state prediction as

$$\hat{H}\left(j,k\right)={\left(D{\left(j,k\right)}^{T}D\left(j,k\right)\right)}^{-1}D{\left(j,k\right)}^{T}\left(P\left(j,k\right)-C\left(j,k\right){\hat{x}}^{-}\left(j,k\right)\right).$$

(4) Updated unconstrained state estimate

$${\hat{x}}^{+}\left(j,k\right)={\hat{x}}^{-}\left(j,k\right)+K\left(j,k\right)\left(P\left(j,k\right)-C\left(j,k\right){\hat{x}}^{-}\left(j,k\right)-D\left(j,k\right)\hat{H}\left(j,k\right)\right).$$ (31)

(5) Error covariance of ${\hat{x}}^{+}\left(j,k\right)$

$${\left(R^{+}\left(j,k\right)\right)}^{-1}={\left(R^{-}\left(j,k\right)\right)}^{-1}+C^{⊺}\left(j,k\right){\left[D\left(j,k\right)QD^{⊺}\left(j,k\right)+R_{n}\right]}^{-1}C\left(j,k\right).$$ (32)

After running the five steps of the unconstrained filter, each cell obtains an unconstrained state estimate ${\hat{x}}^{+}\left(j,k\right)$ , and it will share this information with all neighboring cells to update the constrained estimate.

Since each cell will not shrink to zero size and the flow velocity vector *v* ≠ 0, the following relationship can be always satisfied:

$$\begin{array}{ll} G^{⊺}\left(j,k\right)G\left(j,k\right) & ={\left(-1+\frac{\theta \Delta t}{A\left(C_{j}\right)}\underset{s\in \partial C_{j}}{\sum }\frac{1}{\cos\theta_{s}}\frac{\|r_{i,j}^{k-1}-r_{i^{\prime },j}^{k-1}{\|}_{2}}{\|r_{c_{j}}^{k-1}-r_{c_{j^{\prime }}}^{k-1}{\|}_{2}}\right)}^{2}\\ & \;+{\left(\Delta t\right)}^{2}\|v{\|}^{2}+1\neq 0,\forall j,k. \end{array}$$ (33)

(6) Updated constrained state estimate

$$\begin{array}{ll} {\tilde{x}}^{+}\left(j,k\right) & ={\hat{x}}^{+}\left(j,k\right)-G\left(j,k\right){\left(G^{⊺}\left(j,k\right)G\left(j,k\right)\right)}^{-1}\\ & \;\times \left(G^{⊺}\left(j,k\right){\hat{x}}^{+}\left(j,k\right)-\hat{g}\left(j,k\right)\right), \end{array}$$ (34)

where

$$\begin{array}{cc} \hat{g}\left(j,k\right)≜ & -\frac{\theta \Delta t}{A\left(C_{j}\right)}\underset{s\in \partial C_{j}}{\sum }\tan\theta_{s}\left[p\left(r_{i,j}^{k-1},k-1\right)-p\left(r_{i^{\prime },j}^{k-1},k-1\right)\right]\\ & +\frac{\theta \Delta t}{A\left(C_{j}\right)}\underset{s\in \left(\partial C_{j}⧵\partial \Omega_{r}\right)}{\sum }\frac{1}{\cos\theta_{s}}\frac{\|r_{i,j}^{k-1}-r_{i^{\prime },j}^{k-1}{\|}_{2}}{\|r_{c_{j}}^{k-1}-r_{c_{j^{\prime }}}^{k-1}{\|}_{2}}{\hat{z}}^{+}\left(r_{c_{j^{\prime }}}^{k-1},k-1\right)\\ & +\frac{\theta \Delta t}{A\left(C_{j}\right)}\underset{s\in \left(\partial C_{j}\cap \partial \Omega_{r}\right)}{\sum }\frac{1}{\cos\theta_{s}}\frac{\|r_{i,j}^{k-1}-r_{i^{\prime },j}^{k-1}{\|}_{2}}{\|r_{c_{j}}^{k-1}-r_{c_{j^{\prime }}}^{k-1}{\|}_{2}}\\ & \times \frac{p\left(r_{i,j}^{k-1},k-1\right)+p\left(r_{i^{\prime },j}^{k-1},k-1\right)}{2}. \end{array}$$ (35)

This is an approximation of ***g*** (*j*, *k*) with ${\hat{z}}^{+}\left(r_{c_{j^{\prime }}}^{k-1},k-1\right)=\left[\begin{array}{cccc} 1 & 0_{1\times d} & 0 & 0_{1\times d} \end{array}\right]{\hat{x}}^{+}\left(j^{\prime },k\right)$ , and it uses measurements $p\left(r_{i,j}^{k-1},k-1\right),p\left(r_{i^{\prime },j}^{k-1},k-1\right)$ as approximations for $z\left(r_{i,j}^{k-1},k-1\right),z\left(r_{i^{\prime },j}^{k-1},k-1\right)$ . According to the definition $G\left(j,k\right)={\left[\begin{array}{cccc} \left(-1+\frac{\theta \Delta t}{A\left(C_{j}\right)}\sum_{s\in \partial C_{j}}\frac{1}{\cos\theta_{s}}\frac{\|r_{i,j}^{k-1}-r_{i^{\prime },j}^{k-1}{\|}_{2}}{\|r_{c_{j}}^{k-1}-r_{c_{j^{\prime }}}^{k-1}{\|}_{2}}\right) & -{\left(v\Delta t\right)}^{⊺} & 1 & 0_{1\times d} \end{array}\right]}^{⊺}$ , the updated constrained estimate in Eq. 34 updates the estimates of $z\left(r_{c_{j}}^{k-1},k-1\right),\nabla z\left(r_{c_{j}}^{k-1},k-1\right),z\left(r_{c_{j}}^{k-1},k\right)$ and does not affect the gradient estimate $\nabla z\left(r_{c_{j}}^{k-1},k\right)$ obtained by Eq. 31.

Remark 5.2 For the six steps of the distributed constrained cooperative Kalman filter described in Eqs 28–34, the first five steps in Eqs 28–32 use the local information in one cell, while the last step in Eq. 34 uses estimated information from neighboring cells.

Remark 5.3 The distributed constrained cooperative Kalman filter proposed in Zhang et al. (2023) updates the field value estimate without affecting the gradient estimation, while this work updates the whole state estimate using the constraint information. In addition, the formation cells can be arbitrary shapes and arbitrary sizes as long as the areas are non-zero indicated by Eq. 33.

Define a combined state vector ***x*** (*k*) to include all distributed state vectors as

$$x\left(k\right)={\left[\begin{array}{c} x^{⊺}\left(1,k\right),\ldots ,x^{⊺}\left(N,k\right) \end{array}\right]}^{⊺},$$ (36)

which represents the true state value. Similarly, we can have updated unconstrained combined state estimate ${\hat{X}}^{+}\left(k\right)={\left[\begin{array}{c} {\hat{x}}^{+}{\left(1,k\right)}^{⊺},\ldots ,{\hat{x}}^{+}{\left(N,k\right)}^{⊺} \end{array}\right]}^{⊺}$ and constrained combined state estimate ${\tilde{X}}^{+}\left(k\right)={\left[\begin{array}{c} {\tilde{x}}^{+}{\left(1,k\right)}^{⊺},\ldots ,{\tilde{x}}^{+}{\left(N,k\right)}^{⊺} \end{array}\right]}^{⊺}$ . Then, we can have one constrained cooperative Kalman filter of ***x*** (*j*, *k*) as

$$\begin{array}{cc} {\hat{X}}^{-}\left(k\right) & =A\left(k-1\right){\tilde{X}}^{+}\left(k-1\right)+U\left(k-1\right),\\ R^{-}\left(k\right) & =A\left(k-1\right)R^{+}\left(k-1\right)A^{⊺}\left(k-1\right)+W,\\ K\left(k\right) & =R^{-}\left(k\right)C^{⊺}\left(k\right){\left[C\left(k\right)R^{-}\left(k\right)C^{⊺}\left(k\right)+D\left(k\right)QD^{⊺}\left(k\right)+R_{n}\right]}^{-1},\\ {\hat{X}}^{+}\left(k\right) & ={\hat{X}}^{-}\left(k\right)+K\left(k\right)\left(P\left(k\right)-C\left(k\right){\hat{X}}^{-}\left(k\right)-D\left(k\right)\hat{H}\left(k\right)\right),\\ {\left(R^{+}\left(k\right)\right)}^{-1} & ={\left(R^{-}\left(k\right)\right)}^{-1}+C^{⊺}\left(k\right){\left[D\left(k\right)QD^{⊺}\left(k\right)+R_{n}\right]}^{-1}C\left(k\right),\\ {\tilde{X}}^{+}\left(k\right) & ={\hat{X}}^{+}\left(k\right)-G\left(k\right){\left(G^{⊺}\left(k\right)G\left(k\right)\right)}^{-1}\left(G^{⊺}\left(k\right){\hat{X}}^{+}\left(k\right)-\hat{g}\left(k\right)\right), \end{array}$$ (37)

where ***A*** (*k*) = diag(***A*** (1, *k*), …, ***A*** (*N*, *k*)), ***C*** (*k*) = diag(***C*** (1, *k*), …, ***C*** (*N*, *k*)), $D\left(k\right)={\left[\begin{array}{c} D^{⊺}\left(1,k\right),\ldots ,D^{⊺}\left(N,k\right) \end{array}\right]}^{⊺}$ , $P\left(k\right)={\left[\begin{array}{c} P^{⊺}\left(1,k\right),\ldots ,P^{⊺}\left(N,k\right) \end{array}\right]}^{⊺}$ , $\hat{H}\left(k\right)={\left[\begin{array}{c} {\hat{H}}^{⊺}\left(1,k\right),\ldots ,{\hat{H}}^{⊺}\left(N,k\right) \end{array}\right]}^{⊺}$ , ***G*** (*k*) = diag(***G*** (1, *k*), …, ***G*** (*N*, *k*)), and $\hat{g}\left(k\right)={\left[\begin{array}{c} \hat{g}\left(1,k\right),\ldots ,\hat{g}\left(N,k\right) \end{array}\right]}^{⊺}$ .

Algorithm 1 Distributed constrained cooperative Kalman filter

For each individual cell *C*_*j*_, *j* = 1, …, *N*:
Initialize the constrained state estimate, ${\tilde{x}}^{+}\left(j,0\right)$ ;
Initialize the locations of mobile sensors, ${\left\{r_{i,j}^{1}\right\}}_{i=1,\ldots ,|C_{j}|}$ ;
Initialize time step *k* = 1;
1: **while** true **do**
2: Receive measurements ${\left\{z_{i,j}^{k}\right\}}_{i\in C_{j}}$ from mobile sensors in cell *C*_*j*_ and generate unconstrained state estimation ${\hat{x}}^{+}\left(j,k\right)$ according to Eqs 28–32 (**intra-cell communication**);
3: Communicate the unconstrained state estimation ${\hat{x}}^{+}\left(j,k\right)$ to the neighboring cells of *C*_*j*_ and obtain the constrained state estimate ${\tilde{x}}^{+}\left(j,k\right)$ using Eq. 34 (**inter-cell communication**);
4: Move to the next mobile sensor locations, ${\left\{r_{i,j}^{k+1}\right\}}_{i=1,\ldots ,|C_{j}|}$ ;
5: *k*≔*k* + 1;
6: **end while**

One challenge encountered by the centralized cooperative Kalman filter is the high computation cost for large-scale systems. Our proposed distributed filtering strategy can avoid the problem of high computation costs when applied in large-scale systems. One possible solution is that we split the mobile sensors into two types—“computing, sensing, and communicating” (CSC) mobile sensors and “sensing and communicating” (SC) mobile sensors. CSC sensors take measurements and compute all the Kalman filter estimations, while SC sensors only collect measurements. Each SC sensor is required to share a cell with one CSC sensor. Each communication cell has at least one CSC sensor and multiple SC sensors.

**FIGURE 3:**
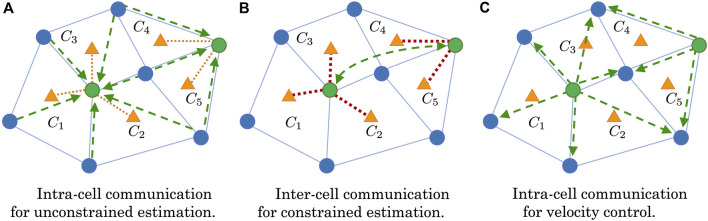
Communication flow of the distributed mobile sensor network during estimation and velocity control. Green dots represent the CSC sensors, while blue dots represent the SC sensors, and orange triangles represent the cell formation centers. Green dashed arrows represent communication or information sharing with direction. Orange and dark red dashed lines represent unconstrained and constrained state estimations, respectively. **(A)** Intra-cell communication for unconstrained estimation. **(B)** Inter-cell communication for constrained estimation. **(C)** Intra-cell communication for velocity control.

The communication and data flow within the distributed mobile sensor network can be described as follows (also shown in Figure 3):

1. All sensors (SC and CSC) move and take a measurement of the field.
2. SC sensors (blue dots) in each cell send their data to their connected CSC sensors (green dots) (Figure 3A).
3. The CSC sensor performs the unconstrained cooperative Kalman filter to estimate the measurement values and gradients at the cell center (orange triangles) for each cell it is connected to.
4. The CSC sensors communicate with their “connected” CSC neighbors to get all the necessary sensor positions, measurements, and cell center estimates. (“connected” CSC neighbors are CSC mobile sensors that share a mobile sensor in their connected cells) (Figure 3B).
5. Each CSC sensor computes the constrained cooperative Kalman filter for each connected cell.
6. Cell estimates are pushed back to the connected mobile sensors for feedback control (Figure 3C).
7. Repeat.

## 6 Formation control design

In this section, we will design a formation controller for the mobile sensor network using the gradient estimation obtained from the distributed constrained cooperative Kalman filter.

Denote the desired distance between two sensors *i*, *i*′ as *d*_*ii*′_. According to the state estimation using the constrained cooperative Kalman filter, we are able to obtain gradient estimation at each cell center. Thus, instead of directly using the gradient of each sensor, we consider utilizing the gradient estimation of cell centers related to the mobile sensor.

If sensor *i* belongs to cell *j*, we write it as *i* ∈ *C*_*j*_. Define $J_{i}=\left\{j|i\in C_{j}\right\}$ as the set that contains all the cell indices that sensor *i* is related to. Then, define $v_{i}^{k}$ as a weighted summation of gradient estimations from connected cell centers:

$$v_{i}^{k}≜\underset{j\in J_{i}}{\sum }\frac{1}{\|r_{i}^{k}-r_{c_{j}}^{k}{\|}_{2}}\nabla z\left(r_{c_{j}}^{k}\right),$$ (38)

which implies that if ***r***_*i*_ is closer to cell center $r_{c_{j}}$ than $r_{c_{j^{\prime }}}$ , then estimation $\nabla z\left(r_{c_{j}}\right)$ has more influence on $v_{i}^{k}$ than $\nabla z\left(r_{c_{j^{\prime }}}\right)$ does. This describes a dynamical way of gradient approximation determined by distances.

By applying the gradient approximation in Eq. 38, the gradient-based desired distance can be designed as follows:

$$d_{ii^{\prime }}=\frac{\epsilon_{1}}{{\left\|\frac{1}{2}\left(v_{i}^{k}+v_{i^{\prime }}^{k}\right)\right\|}_{2}+\epsilon_{2}}+\epsilon_{3}\in \left(\epsilon_{3},\frac{\epsilon_{1}}{\epsilon_{2}}+\epsilon_{3}\right),$$ (39)

where $\epsilon_{1},\epsilon_{2},\epsilon_{3}\in R_{+}$ are tuning parameters.

The desired distances between two communicating sensors Eq. 39 are designed to be gradient-dependent. Moreover, if the norm of the approximated gradient increases, the desired distance will decrease, which indicates that the formation size will shrink while encountering an area of larger field variation. In this way, a smaller formation will provide more accurate state estimation by collecting closer sensor measurements and can avoid ignoring large variations within a small domain. When the sensors are exploring a field with decreasing gradient norm, larger distances can still ensure the accuracy of state estimation due to the slow field variation. Both cases demonstrate the ability of the proposed gradient-based adaptive formation to be suitable for various scenarios, such as source-seeking and contour mapping (Han and Chen, 2014), where the rigid formation fails to provide accurate estimation for a field with a large variation or the field is too simple for the capability of the deployed sensors. In this paper, we are focusing on the application of source-seeking using distributed mobile sensor networks. Additionally, the tuning parameters in Eq. 39 ensure the boundedness of the desired distances so that the formation will be controllable, not shrinking to zero size or becoming too large.

Since the mobile sensors are taking discrete measurements, we will apply discrete time control written as follows:

$$r_{i,j}^{k+1}=r_{i,j}^{k}+u_{i}^{k}\Delta t,$$ (40)

where Δ*t* is the sampling time and is set to be constant. Define $w=\frac{\nabla z+e}{\|\nabla z+e{\|}_{2}}$ as the unit length direction vector along the gradient direction ∇*z* perturbed by some estimation error *e*.

We approximate the gradient direction at ***r***_*i*_ using the gradient estimations at all the cell centers around it as

$$w_{i}^{k}=\frac{v_{i}^{k}}{\|v_{i}^{k}{\|}_{2}}=\frac{\sum_{j\in J_{i}^{v}}\frac{1}{\|r_{i}-r_{c_{j}}{\|}_{2}}\nabla z\left(r_{c_{j}}^{k}\right)}{{\left\|\sum_{j\in J_{i}^{v}}\frac{1}{\|r_{i}-r_{c_{j}}{\|}_{2}}\nabla z\left(r_{c_{j}}^{k}\right)\right\|}_{2}}.$$ (41)

The control input for sensor *i* is designed as

$$u_{i}^{k}=-aw_{i}^{k}+b{\left(w_{i}^{k}\right)}^{⊥}+cf_{i}^{k},$$ (42)

where *a*, *b*, *c* are tuning parameters and ${\left(w_{i}^{k}\right)}^{⊥}$ is the normal unit vector of $w_{i}^{k}$ . The formation terms $f_{i}^{k}$ are defined as

$$f_{i}^{k}=\underset{i^{\prime }\neq i}{\sum }\frac{\|r_{i^{\prime }}^{k}-r_{i}^{k}{\|}_{2}-d_{ii^{\prime }}^{k}}{\|r_{i^{\prime }}^{k}-r_{i}^{k}{\|}_{2}}\left(r_{i^{\prime }}^{k}-r_{i}^{k}\right),$$ (43)

where $d_{ii^{\prime }}^{k}$ is a desired separation distance between sensors *i* and *i*′. The formation term enables the mobile sensors to maintain formations close to the desired ones, by letting sensors move away from each other if the actual distance is larger than the desired one and letting sensors move closer if the actual distance is smaller.

Remark 6.1 The control input $u_{i}^{k}$ defined in Eq. 42 is designed for source-seeking, and it enables the sensors to follow the gradient direction and reach the source, which is the main application considered in this paper. In addition to source-seeking, mobile sensors can also be deployed for other applications, such as contour mapping, where sensors are required to follow the normal direction of the gradient. The control input in Eq. 42 can be modified based on the speeding-up and slowing-down (SUSD) strategy (Wu and Zhang, 2015; Al-Abri et al., 2018; Zhang et al., 2020).

$$u_{i}^{k}=-a\left(p_{i}^{k}-z_{d}\right)w_{i}^{k}+b{\left(w_{i}^{k}\right)}^{⊥}+cf_{i}^{k},$$ (44)

and the only change is the multiplication of $\left(p_{i}^{k}-z_{d}\right)$ , where $p_{i}^{k}$ is the noisy measurement of the field taken by agent *i* at the *k* *th* time step and *z*_*d*_ is the field value of the desired level.

## 7 Convergence analysis

In this section, convergence analysis for the formation control and the distributed constrained cooperative Kalman filter will be provided.

Theorem 7.1 Under the formation control (Eq. 42) along with (Eq. 43) and (Eq. 39), the mobile sensors can always maintain bounded formations with bounded speed.

Proof In Oh et al. (2015), the controller defined by Eq. 43 is a well-understood control law that is guaranteed to achieve the formation specified by the desired constant inter-agent distances. Since $d_{ii^{\prime }}^{k}\in \left(\epsilon_{3},\frac{\epsilon_{1}}{\epsilon_{2}}+\epsilon_{3}\right)$ is bounded by Eq. 39, the formation can be achieved with bounded error. Then, there exists $\epsilon_{4}\in R_{+}$ such that for any two neighboring sensors,

$$\|r_{i^{\prime }}^{k}-r_{i}^{k}{\|}_{2}-d_{ii^{\prime }}^{k}\in \left[-\epsilon_{4},\epsilon_{4}\right].$$ (45)

This implies that

$$\|r_{i^{\prime }}^{k}-r_{i}^{k}{\|}_{2}\in \left[\epsilon_{3}-\epsilon_{4},\frac{\epsilon_{1}}{\epsilon_{2}}+\epsilon_{3}+\epsilon_{4}\right],$$ (46)

where the parameter *ϵ*_3_ can be chosen such that *ϵ*_3_ > *ϵ*_4_.

Since the formation is bounded by Eq. 45, the formation term satisfies

$$\begin{array}{ll} \|f_{i}^{k}{\|}_{2} & ={\left\|\underset{i^{\prime }\in N_{i}}{\sum }\frac{\|r_{i^{\prime }}^{k}-r_{i}^{k}{\|}_{2}-d_{ii^{\prime }}^{k}}{\|r_{i^{\prime }}^{k}-r_{i}^{k}{\|}_{2}}\left(r_{i^{\prime }}^{k}-r_{i}^{k}\right)\right\|}_{2}\\ & \leq \underset{i^{\prime }\in N_{i}}{\sum }{\left\|\frac{\|r_{i^{\prime }}^{k}-r_{i}^{k}{\|}_{2}-d_{ii^{\prime }}^{k}}{\|r_{i^{\prime }}^{k}-r_{i}^{k}{\|}_{2}}\left(r_{i^{\prime }}^{k}-r_{i}^{k}\right)\right\|}_{2}\leq \underset{i^{\prime }\in N_{i}}{\sum }\epsilon_{4}. \end{array}$$ (47)

According to the discrete time control law in Eq. 42, the norm of control input $u_{i}^{k}$ satisfies

$$\begin{array}{ll} \|u_{i}^{k}{\|}_{2} & =\|-aw_{i}^{k}+b{\left(w_{i}^{k}\right)}^{⊥}+cf_{i}^{k}{\|}_{2}\leq \|aw_{i}^{k}{\|}_{2}+\|b{\left(w_{i}^{k}\right)}^{⊥}{\|}_{2}\\ & \;+\|cf_{i}^{k}{\|}_{2}\leq |a|+|b|+|c|\underset{i^{\prime }\in N_{i}}{\sum }\epsilon_{4}, \end{array}$$ (48)

where the last inequality can be obtained from Eq. 47 and $\|w_{i}^{k}{\|}_{2}=\|{\left(w_{i}^{k}\right)}^{⊥}{\|}_{2}=1$ .

Therefore, the mobile sensors are capable of maintaining bounded formation with bounded speed by Eqs 46, 48.

The information dynamics and measurement equation considered for each individual cell in this paper share the same structure as those in Zhang et al. (2022), where a centralized cooperative Kalman filter has been proposed with provable convergence.

Proposition 7.2 [Propositions VI.4 and VI.6 in Zhang et al. (2022)] The state dynamics (13) with the measurement equation (17) are uniformly completely controllable and uniformly completely observable if the following conditions are satisfied: (Cd1) The covariance matrix ***W*** is bounded, i.e., *λ*_1_ ***I*** ≤ ***W*** ≤ *λ*_2_ ***I*** for some constants *λ*_1_, *λ*_2_ > 0. (Cd2) The speed of each agent is uniformly bounded, i.e., $\|r_{i,j}^{k}-r_{i,j}^{k-1}{\|}_{2}\leq \lambda_{3}$ for all *i*, *j*, *k*, and some constant *λ*_3_ > 0. (Cd3) The number of sensors in one cell *C*_*j*_ satisfies $|C_{j}|>\frac{d^{2}}{2}$ for all *j*. (Cd4) The covariance matrices ***R***_*n*_ and ***Q*** are bounded, i.e., *λ*_4_ ***I*** ≤ ***R***_*n*_ ≤ *λ*_5_ ***I*** and 0 ≤ ***Q*** ≤ *λ*_6_ ***I*** for some *λ*_4_, *λ*_5_, *λ*_6_> 0. (Cd5) The distance between each agent and the cell center is uniformly bounded from both above and below, i.e., $\lambda_{7}\leq \|r_{i,j}^{k-1}-r_{c_{j}}^{k-1}\|\leq \lambda_{8}$ for all *i*, *j*, *k*, and some *λ*_7_, *λ*_8_ > 0. (Cd6) There exists a constant time difference *τ*_2_, and for all *k* > *τ*_2_, there exists a time instance *k*_1_ ∈ [*k* − *τ*_2_, *k*], as well as two sets of agents indexed by {*i*_1_, …, *i*_*d*_}, {*i*_*d*+1_, …, *i*_2*d*_}, respectively, such that $\left(r_{i_{1},j}^{k_{1}-1}-r_{c_{j}}^{k_{1}-1}\right),\ldots ,\left(r_{i_{\text{d}},j}^{k_{1}-1}-r_{c_{j}}^{k_{1}-1}\right)$ are linearly independent, and $\left(r_{i_{d+1,j}}^{k_{1}}-r_{c_{j}}^{k_{1}-1}\right),\ldots ,\left(r_{i_{2d,j}}^{k_{1}}-r_{c_{j}}^{k_{1}-1}\right)$ are linearly independent.

Remark 7.3 In Proposition 7.2, (Cd2) and (Cd5) are guaranteed by bounded sensor speed and bounded formation cells under (Eq. 40) along with (Eq. 43).

Since the unconstrained cooperative Kalman filter is both uniformly completely controllable and observable (Proposition 7.2), the unconstrained filter for each individual cell is convergent. This means that $\left\|x_{j}-{\hat{x}}_{j}\right\|$ is bounded for all *j*, where *x*_*j*_ represents the true state value.

Since the combined filter is constructed by stacking all distributed filters together and the unconstrained filter for each individual cell is convergent, the combined unconstrained filter is also convergent.

$$\|X-\hat{X}{\|}_{2}=\sqrt{\overset{N}{\underset{j=1}{\sum }}\|x-x_{j}{\|}_{2}^{2}}\leq \overset{N}{\underset{j=1}{\sum }}\|x-x_{j}{\|}_{2}.$$ (49)

From Theorem 4 in Simon and Chia (2002), since the unconstrained combined cooperative Kalman filter is convergent, the constrained combined cooperative Kalman filter is also convergent. Thus, each distributed constrained cooperative Kalman filter is convergent.

## 8 Simulation results

In this section, simulation results using a distributed mobile sensor network of 12 sensors will be presented to validate that the proposed distributed algorithm enables mobile sensor networks to estimate information at each cell center along trajectories of a collection of cell centers. According to the 1D advection–diffusion solution in Mojtabi and Deville (2015), we consider a 2D advection–diffusion equation as $z\left(r,t\right)=-\sin\left(\pi \left(k_{1}r_{x}+v_{1}k_{2}t\right)\right)e^{-\theta \pi^{2}k_{2}t}-\sin\left(\pi \left(k_{1}r_{y}+k_{2}v_{2}t\right)\right)e^{-\theta \pi^{2}k_{2}t}$ and $r={\left[r_{x},r_{y}\right]}^{⊺}$ , with diffusion coefficient *θ* = 2 and flow velocity ***v*** = [0.5,1]^⊺^, *k*_1_ = 0.05, *k*_2_ = 0.0001, and the SNR for the field is set to be 10. The sample rate is 1 Hz, and the simulation of 12 sensors will be running for 600 s.

**FIGURE 4:**
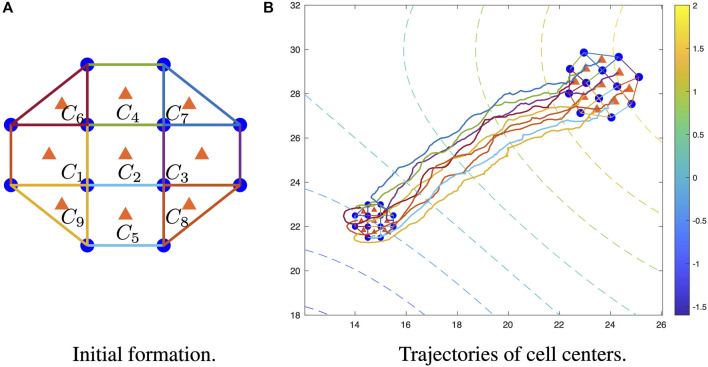
Trajectory of cell centers in a mobile sensor network. **(A)** Initial 9-cell formation shape of a 12-sensor network. **(B)** Trajectories of cell centers with initial formation (bottom left smaller one) and final formation (upper left larger one), where the colored dashed lines represent the level curve of the field. For simulation videos, please see https://youtu.be/aHrvXjt8u4M (same initial location), and https://youtu.be/8_gvyacW-5g (different initial locations).

At each time step, mobile sensors will take noisy measurements of the field, and the sensor locations are also available. Based on the network structure of the mobile sensors (Figure 4), three or four sensor measurements will be incorporated for estimation within each cell. The estimation for field value and gradient at each cell center is composed of two parts: unconstrained and constrained estimations. In order to complete unconstrained estimation (Eqs. 28–32), noisy measurements, previous and current sensor locations, and previous estimations pertaining to each cell are required. Then, the constrained estimation can be obtained by projecting the unconstrained estimation onto the constrained space (Eq. 26) defined by the PDE field, where information sharing of unconstrained estimation between neighboring cells is required to have the PDE constraint.

As shown in Figure 4A, the mobile sensors have been organized into four triangle cells and five rectangle cells, where the blue dots represent the mobile sensors, the orange triangles represent cell centers, and the blue lines represent the edges of each individual cell. The shape of each cell is fixed due to the predetermined communication graph by Assumption 2.1, and there is no requirement for the cell shapes to be symmetric. The five rectangles in the initial formation are symmetric, while the four triangles are asymmetric.

In Figure 4B, the dashed contours represent the level curves of the field values, and the colored lines are the trajectories of the nine cell centers. The smaller formation on the bottom left is the initial location, and the larger formation on the upper right is the final location. Driven by Eq. 42, the mobile sensors will adjust the distances between neighbors according to the norms of estimated gradients, which leads to non-rigid gradient-based formation for the mobile sensor networks and also explains the size difference between initial and final formations.

**FIGURE 5:**
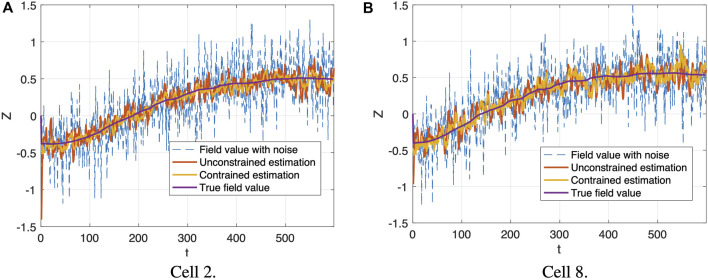
Field estimation generated by distributed constrained cooperative Kalman filter (yellow lines) at the cell center of cell 2 and cell 8 along trajectories, compared with field value with noise (blue dashed lines), distributed unconstrained cooperative Kalman filter estimation (red lines) and true field value (purple lines). The *x*-axis is time *t*, and the *y*-axis is the field value *z*(***r***, *t*). **(A)** Cell 2. **(B)** Cell 8.

The field estimations generated by the proposed distributed constrained filtering at the cell centers of cell 2 and cell 8 along trajectories are provided in Figure 5. The noisy field values indicated by the blue dashed lines are relatively inaccurate, compared with the true field values marked by purple solid lines. Both the unconstrained and constrained filtering strategies (marked by red and yellow, respectively) are capable of reducing the noise. These four lines in each plot of Figure 5 share the same trajectory. At each time step, we run the distributed constrained cooperative Kalman filter in Eq. 37 for each cell, and then the formation control in Eq. 42 is updated based on the estimated gradient information. At the same time, a distributed unconstrained filter is also running for each cell to obtain an unconstrained state estimation, but such estimation will not be used to update the formation control. The constrained one shows improved performance, which demonstrates that more accurate estimation can be generated when more field information is incorporated by adding PDE constraints. We also observe the differences in estimation performance among cells, as shown by the two example cells in Figure 5. According to Figure 4A, cell 2 is the center cell of the formation, and it has eight neighboring cells, which provide information for updated constrained state estimation, while cell 8 only has three neighboring cells. This explains why when comparing with unconstrained estimations, the constrained estimations in cell 8 show less improvement than the constrained estimations in cell 2.

Statistical data of estimation errors at cell centers $r_{c_{1}},\ldots ,r_{c_{9}}$ are provided in Table 1 along with the corresponding data of measurement noise. The subscripts *n*, *u*, *c* represent measurement noise, unconstrained estimation error, and constrained estimation error, respectively. The first row denotes the nine cells. Rows 2–4 represent the means of the errors between true field value and noise, unconstrained estimation, and constrained estimation, respectively. The last three rows represent the standard deviations of the errors between true field value and noise, unconstrained estimation, and constrained estimation, respectively.

**TABLE 1:** Statistical data of state estimation errors and noise.

	Cell *C*_1_	Cell *C*_2_	Cell *C*_3_	Cell *C*_4_	Cell *C*_5_	Cell *C*_6_	Cell *C*_7_	Cell *C*_8_	Cell *C*_9_
*mean*_*n*_	0.0028	0.0075	−0.0214	−0.0019	−0.0085	0.0205	0.0047	0.0054	0.0007
*mean*_*u*_	−0.0001	−0.0021	−0.0033	0.0087	−0.0092	0.0002	0.0014	−0.0003	−0.0106
*mean*_*c*_	−0.001	−0.0125	−0.0103	0.0025	−0.0218	−0.0083	0.0061	0.0038	0.0002
*std*_*n*_	0.3115	0.3248	0.3217	0.3257	0.3164	0.3166	0.3309	0.3169	0.2997
*std*_*u*_	0.105	0.1004	0.1054	0.1026	0.1043	0.1144	0.1071	0.1201	0.1145
*std*_*c*_	0.0711	0.0555	0.0722	0.0685	0.0789	0.0788	0.0747	0.0933	0.0871

According to Table 1, both unconstrained and constrained filtering improved the estimation of *z*(***r***, *t*), and the constrained filtering had a lower standard deviation than unconstrained filtering in all cells. This validates that more accurate estimation can be generated when more field information is provided.

## 9 Conclusion and future work

In this paper, we proposed a distributed cooperative Kalman filter constrained by the advection–diffusion equation, which solves the estimation problem using a large number of mobile sensors in a distributed way. Both convergence analysis and simulation results demonstrate the effectiveness of our proposed filtering strategy, and the mobile sensors can maintain gradient-based formation. For the next step, we will generalize our method to higher-order PDEs and incorporate parameter identification for the PDE models.

## Data Availability

The original contributions presented in the study are included in the article/Supplementary Material; further inquiries can be directed to the corresponding author.
